# End-stage kidney diseases in areas of conflict: patients’ perspective and patient access to hemodialysis services in Northwest Syria

**DOI:** 10.1186/s12913-025-12673-1

**Published:** 2025-05-02

**Authors:** Ismail Alkhatib, Sami Alasfar, Gilbert Burnham, Nimetcan Mehmet Orhun

**Affiliations:** 1 Public Health Department, Faculty of Medicine, Ankara Yıldırım Beyazıt Üniversitesi, Ankara, Türkiye; 2 Department of Medicine, Division of Nephrology, Mayo Clinic Arizona, 5777 E Mayo Blvd, Phoenix, AZ 85054 USA; 3 Department of International Health, The Johns Hopkins Bloomberg School of Public Health, 615 N Wolfe Street, Baltimore, MD 21205 USA; 4 Global Health Department, Public Health Institute, Ankara Yıldırım Beyazıt Üniversitesi Çubuk Yerleşkesi, Ankara, Türkiye

**Keywords:** End-stage kidney disease, Hemodialysis, Conflict zones, Northwest Syria, Healthcare access, Patients’ perspectives

## Abstract

**Background:**

There are an estimated 850 persons with End-Stage Kidney Disease (ESKD) receiving hemodialysis in the conflict-affected Northwest Syria. This study examines patients’ perspectives, and experiences with hemodialysis and their knowledge about their disease and treatment.

**Methods:**

This study used telephone interviews with 101 randomly selected ESKD patients from 12 of the 14 hemodialysis units in Northwest Syria during early 2023 in a cross-sectional study.

**Results:**

The mean age of respondents was 50.3 ± 16.7 years (range 7 to 81), with 55.4% males. The sample included 53 local residents and 48 Internally Displaced Persons (IDPs) from elsewhere in Syria. A quarter (28.7%) of patients reported being unable to access one or more hemodialysis sessions in the previous year. In the past year, 61 of 101 dialysis patients had changed household location because of reported insecurity. Frequent household relocations disrupted dialysis continuity resulting in the use of multiple dialysis sites. Because of the decentralized distribution of facilities, half of patients could reach their dialysis facility in 30 min or less, and at minimal costs. Two-thirds (67.3%) reported the presence of comorbidities, with hypertension and diabetes being the most common. Of the 35 patients with diabetes, 15 required insulin. Only about half (52.5%) had seen a nephrologist or physician in the past six months. The health literacy level concerning ESKD, and hemodialysis was low for many patients.

**Conclusion:**

While considerable resilience is seen among hemodialysis patients in conflict areas of Northwest Syria, there are deficits in care and health literacy concerning ESKD and hemodialysis which should be addressed. There are a number of low resource actions which could be undertaken for this population to improve their health and understanding of their disease which are currently being considered.

**Supplementary Information:**

The online version contains supplementary material available at 10.1186/s12913-025-12673-1.

## Introduction

As conflicts increasingly involve middle income countries, patients with non-communicable diseases (NCDs) make up an increasingly large component of patients needing services. NCDs pose a demand on outpatient care that primary health care (PHC) services have difficulty in meeting, and referral services often lack sufficient specialist capacity to meet [1, 2]. The increasingly protracted nature of forced displacement often means that NCD services once established may be difficult to financially sustain. In developing plans for NCD services in humanitarian crises, patients with end stage kidney disease (ESKD) receiving hemodialysis dialysis are often considered late. The complexity of ESKD may not be fully appreciated [2]. Hemodialysis services provided to a population are complex and easily disturbed by insecurity, active conflict or natural hazards [3]. Equipment, supplies and staffing may be difficult to maintain or replace. Humanitarian organizations responding to crises are seldom equipped to support the complexities of dialysis services and continuity requirements. This problem has recently gained attention [3]. 

In the 12 years of the ongoing Syrian civil war, millions of Syrians have become refugees or internally displaced persons [4, 5]. The conflict has inflicted extensive damage on the healthcare infrastructure, creating challenges in providing care for patients with NCDS such as ESKD [6]. Prior to the onset of the conflict in 2010, the Aleppo, Idlib, and Homs provinces in Syria had a total of 44 dialysis facilities [6]. As the conflict reached these areas, some facilities were destroyed and for others access by patients was lost. At one facility, half of the 35 patients receiving care in 2011 died [7, 8].

**Fig. 1:**
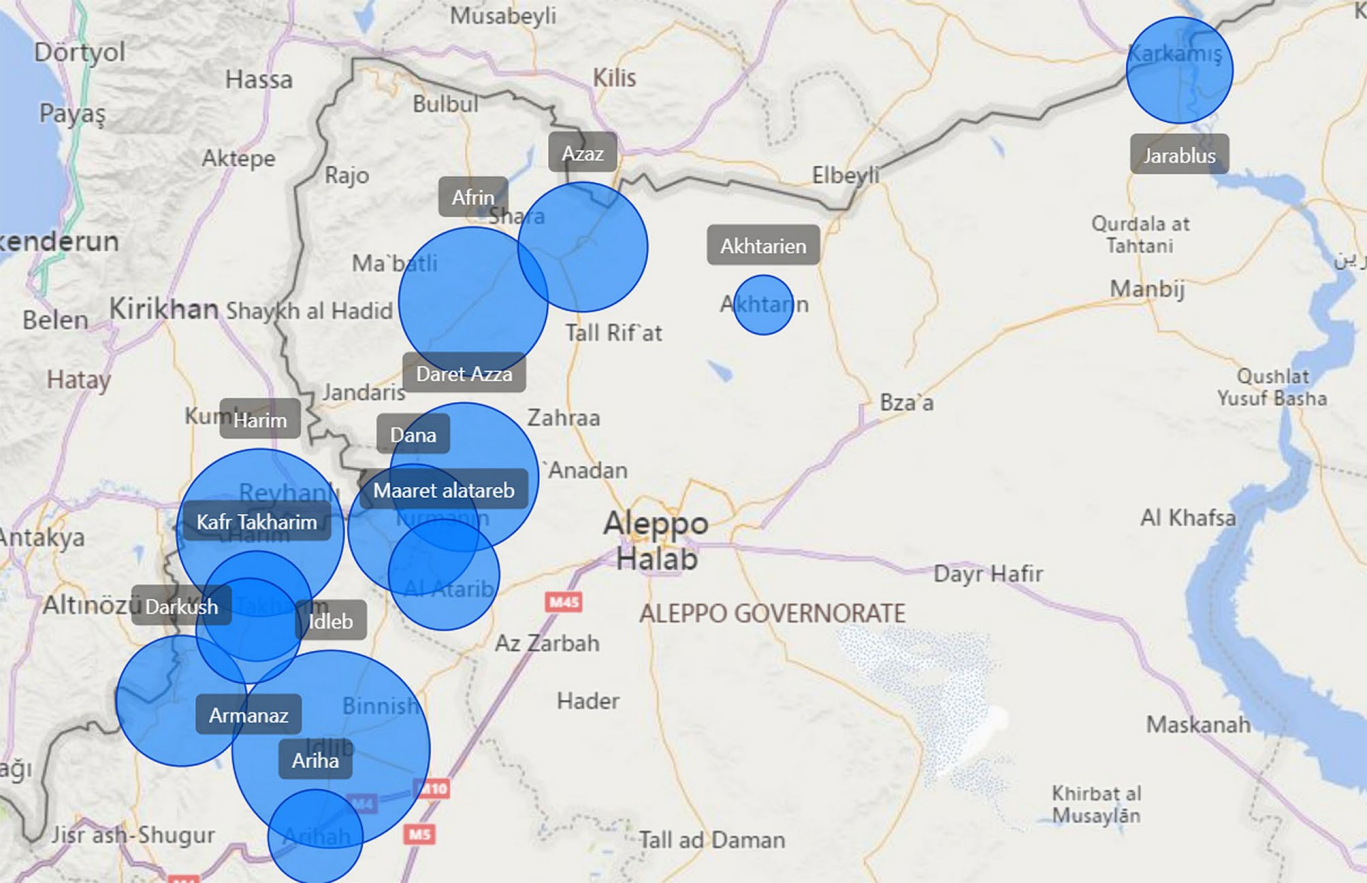
Geographic locations and relative size of hemodialysis centers in NW Syria [10]

Many persons were displaced due to the conflict, fleeing to the opposition-controlled area of Northwest Syria, swelling the population to approximately 4.1 million persons [4, 9] (Fig. 1). There are currently an estimated 850 persons receiving hemodialysis in 14 facilities ranging from hospitals to small community centers [3]. At the end of 2023, there was only one pediatric nephrologist and three adult nephrologists responsible for overseeing the dialysis in Northwest Syria.

The WHO Health Cluster in Gaziantep, Türkiye, as well as several nongovernmental organizations (NGOs) have supported hemodialysis with supplies and equipment. A hemodialysis taskforce was composed to coordinate improvement and supervision. The WHO Heath Cluster undertook a project in 2021 to improve infection prevention and control and medical quality in these facilities in collaboration with Johns Hopkins University and working with the hemodialysis taskforce [3]. The initial assessment of services and establishing a framework of effective care to improve hemodialysis quality in NW Syria have been described in detail elsewhere [10, 11]. In parallel to the hemodialysis technical assessment and quality improvement project, a patient survey was conducted to gain an understanding of access to care, identify treatment barriers encountered and to examine knowledge regarding hemodialysis. In this paper we report the findings of a patient survey.

## Methods

### Study design

This was a cross-sectional study, conducting telephone interviews with a systemic random sample of patients undergoing regular hemodialysis from 5 hemodialysis units in the Northern Aleppo and 7 units in the Idleb Provinces of Northwest Syria. Consent to participate was first obtained from 12 dialysis facilities. Five other facilities declined to participate. From each facility, 10 patients were randomly selected. This constituted 116 patients for the total patient population of 620 (18.7%). Full details of participating facilities, and the questionnaire are found in the supplementary materials. Each dialysis patient provided verbal consent to participate before being interviewed. Survey information is found in the additional materials.

### Data collection

The questionnaire consisted of 37 questions reviewed by the hemodialysis taskforce and translated into Arabic. It was pretested and modifications made as required. Data were collected by telephone after verbal consent from the patient or their caregiver. The data collection took place from February 23rd to March 20th, 2023. Of the 116 preselected patients, only 106 were able to complete the survey because of connectivity difficulties. The telephone interview time averaged 25–30 min. There were no incentives to participate, and no patient contacted declined to participate.

### Data analysis and interpretation

Data was analyzed using the Statistical Package for Social Sciences (SPSS) for Windows version 26. Descriptive statistics such as frequencies and percentages were computed to describe the study population in relation to relevant variables. Bivariate analysis at *p*-value < 0.05 was conducted to assess the correlation between the independent variables and the dependent variable. All data were collected de-identified and stored in a password protected computer file.

## Results

### Sociodemographic characteristic of the participants

The characteristics of the health facilities and the numbers included in the sample are in supplemental materials, Table S1.

The sociodemographic factors and medical comorbidities of study’s 101 participants are presented in Table 1. The mean age was 50.3 (CI 67.0, 33.6) ranging 7–81 years. Two patients (2%) were under age 17. There were 45 (44.6%) females and 56 (55.4%) males. Of all patients, there were 61 (60.4%) who had been forced to move at least once in the previous year due to concerns about security. There were 96 (95%) reported at least one co-morbidity. Hypertension was present in 88 (87.1%) and diabetes in 35 (34.7%).

**Table 1:** Socio-Demographic characteristics of respondents (*N* = 101)

Variable	Frequency (%)
**Age group/ years**	
0–17	2 (2.0)
18–35	22 (21.8)
36–49	19 (18.8)
50–65	42 (41.6)
Above 65	16 (15.8)
**Sex**	
Female	45 (44.6)
Male	56 (55.4)
**Marital status**	
Divorced	3 (3.0)
Married	86 (85,1)
Single	12 (11.9)
**Education level**	
Post secondary	11 (10.9)
Secondary (High School)	14 (13.9)
Primary (Elementary)	48 (47.5)
Illiterate	28 (27.7)
**Resident status**	
Internally Displaced Person (IDP)	48 (47.5)
Local Resident	53 (52.5)
**Number of relocations in past year due to insecurity**	
0	40 (39.6)
1	13 (12.9)
2	19 (18.8)
3	12 (11.9)
4	8 (7.9)
More than 4	9 (8.9)
**Working Status**	
No	80 (79.2)
Yes	21 (20.8)
**Persons earning money regularly in the family**	
No one	27 (26.7)
One person	48 (47.5)
Two Persons	17 (16.8)
Three or more	9 (5.9)
**Comorbidities**	
No comorbidities	5 (5)
One comorbidity	34 (33.7)
Two comorbidities	37 (36.6)
Three or more	25 (24.8)
**Medical Condition**	
High Blood Pressure	88 (87.1)
Diabetes, insulin	15 (14.9)
Diabetes, no Insulin	20 (19.8)
Heart Disease	26 (25.7)
Stroke	7 (6.9)
Others	30 (29.7)

### Disease duration and comorbidities

When dialysis patients were asked how long they had been receiving hemodialysis there were 29 (28.7%) who had been receiving dialysis for a year or less. There were 54(53.4%) who had been receiving dialysis for 1–5 years, and 18 (17.9%) had been on dialysis for more than five years, with a range up to 19 years on dialysis.

### Accessibility to hemodialysis and utilization pattern of hemodialysis patients

More than a quarter (28.7%) of respondents missed scheduled dialysis sessions at least once in the past year (Table 2). There were 7 (7%) who experienced a lack of access at least once in a month. Concerns about security were reported as the single most common cause of missed dialysis sessions. The dialysis clinic itself being closed because of insecurity was commonly reported. Seeking dialysis from another center when a session was missed was common alternative in such cases. There were 76 (75.2%) of respondents undergoing dialysis twice a week, 18 (17.8%) thrice weekly and 7 (6.9%) once a week. Two-thirds of the surveyed patients (67.3%) consistently received dialysis at a single location in the past year, and others had used two locations (20.8%) or three (11.9%).

**Table 2:** Access to Hemodialysis services and utilization pattern of Hemodialysis

Variable	Frequency (%)
**Number of time times the usual dialysis unit was inaccessible in the past year**	
Yes	29 (28.7)
No	72 (71.3%)
**Frequency of inaccessible times**	
No inaccessible encountered	72 (71.3)
Once a week	2 (2)
Once a month	5 (5)
Once every few months	9 (8.9)
Less than once every few months / Less often than above	13 (12.9)
**Reasons for inaccessibility**	
Patient-related factors	22 (51.2)
Dialysis unit-related factor	21 (48.8)
**Handling inability to access the dialysis unit**	
Contact the center to make up for a session later	1 (1)
Do nothing, wait for the center to restart	10 (9.9)
Go to another dialysis center	71 (70.3)
I don’t know what to do	4 (4)
I talk to my relatives to take me to another center	3 (3)
Rent a car to go to another center	9 (8.9)
Walk to another dialysis center	3 (3)
**Number of weekly dialysis session**	
Once	7 (6.9)
Twice	76 (75.2)
Three times	18 (17.8)
**Usual dialysis locations over the past year**	
One location	68 (67.3)
Two locations	21 (20.8)
Three locations	12 (11.9)
**Reasons for changing to the current location**	
No change	68 (67.3)
Another unit more convenient	13 (12.9)
COVID 19	1 (1)
Crowding	2 (2)
Dialysis unit closed	8 (7.9)
Household moved for other reasons	4 (4)
Household moved due to conflict	5 (5)
**Duration of dialysis at current unit**	
Less than 1 year	8 (7.9)
1–2 year	37 (36.6)
2–5 Years	50 (49.5)
More than 5 Years	6 (5.9)
**Total**	101 (100)

### Transportation patterns and cost to access dialysis services

Half of dialysis patients (52.5%) travelled 30 min or less for treatment, and only 9 persons reported more than 60 min (Table 3). One third used their car or motorcycle (35.6%) to reach the dialysis unit and another third (34.7%) relied on Civil Defense vehicles. Over half (57.4%) had no immediate out of pocket transportation costs to reach their usual dialysis center.

**Table 3:** Transportation patterns, cost and access to dialysis services

Variable	Frequency
**Duration to current dialysis site from home**	
Less than 30 min	53 (52.9)
30–59 min	39 (38.6)
60–120 min	7 (6.9)
More than 2 h	2 (2.0)
**Transportation method to the current dialysis site**	
Using public transportation (public transport bus / taxi)	10 (9.9)
Civil defense vehicle	35 (34.7)
Dialysis center vehicle	15 (14.9)
Friend or other drives me	4 (4.0)
My car/ motorcycle	36 (35.6)
Walking	1 (1)
**Were there transportation costs?**	
No	58 (57.4)
Yes	37 (36.6)
Varies—sometimes	6 (5.9)
Transportation fees paid (Turkish Lira)*	
No Fees	58 (57.4)
Less than 50	10 (9.9)
50–150	23 (22.8)
More than 150	10 (9.9)
**Estimated travel duration to next nearest unit**	
less than 30 min	12 (11.9)
30–59 min	38 (37.6)
60–120 min	49 (48.5)
More than 2 h	2 (2.0)
**Total**	101 (100)

*1 USD = 18.5 TL at the time of study

### Medical care received

During the last dialysis clinic visit, more than 90% of patients were weighed and had their blood pressure taken. Patients also reported the following actions during their last dialysis session: health counselling (71.3%), chest auscultation (57.4%), examination for pedal edema (65.3%).

### Health literacy and knowledge about dialysis

Patients were queried about knowledge concerning their ESKD and dialysis. A series of options were posed to patients to assess their understanding of the dialysis process and care of their fistula or port. Only 32.7% were fully literate about the disease questions. Concerning their dialysis, 42.6% were uncertain about some aspects of dialysis and 24.8% held multiple misconceptions.

### Most recent medical consultation

About half (52.5%) of the patients interviewed reported a medical consultation within the last six months (Table 4). A quarter (29.7%) reported a medical consultation more than a year ago but less than 2 years ago, while (6.9%) had their last checkup with a medical doctor more than 2 years ago. Four (4%) don’t remember when their last doctor’s consultation took place. The findings reported during those medical consultations are shown in Table 4.

**Table 4:** Frequency of medical checkups and medical advice reportedly received

Variable	Frequency (%)
**Last doctor’s checkup**	
During last 6 months	53 (52.5)
During last year but no less than 6 months	7 (6.9)
More than a year ago	30 (29.7)
More than 2 years ago	7 (6.9)
Don’t remember	4 (4.0)
**Doctor’s feedback on dialysis treatment**	
Don’t remember	31 (30.7)
Nothing	21 (20.8)
Specifics Feedback	49 (48.5)
**Specifics feedback**	
Add/ Modify medications	10 (9.9)
Continuing blood pressure monitoring	2 (2.0)
Diet must be followed to avoid deterioration	3 (3.0)
Discussing Kidney transplantation	6 (5.9)
Kidney atrophy	2 (2.0)
Need dialysis sessions for life	7 (6.9)
Re-analysis request	5 (5.0)
Reducing dialysis sessions to once a week	1 (1.0)
Request additional medical investigations	5 (5.0)
The patient’s condition is deteriorating	3 (3.0)
The situation is improving and stabilizing	5 (5.0)
**Total**	101 (100)

### Factors associated with experiencing inaccessibility to dialysis services

The factors associated with experiencing inaccessibility to dialysis services are shown in Table 5. There is a significant association between education level and inaccessibility to dialysis units (*p* = 0.024). There is also a statistically significant association between utilizing services from multiple dialysis units and the likelihood of experiencing inaccessibility (*p* = 0.002). Similarly, the number of regular dialysis locations used over the past year (*p* = 0.007). Also, the residency status (*p* = 0.022), relocating due to reported insecurity (*p* = 0.014), and the frequency of residency relocation due to insecurity (*p* = 0.046) showed a significant statistical association with inaccessibility to dialysis services. Binary logistic analysis of association between the significant variables and the occurrence of inaccessibility to dialysis units showed that patients who moved due to perceived insecurity are 4.5 times more likely to experience inaccessible times to the dialysis unit compared to those who did not move, (*p* = 0.012) and 95% CI (1.39–14.47). The patients who utilized hemodialysis services from several units are 3.8 times more likely to experience inaccessible times to the dialysis unit (*p* = 0.009), and 95% CI (1.40- 10.44). In contrast, the analysis of education levels did not show statistically significant differences for individuals across all education categories, including secondary, primary, and illiterate. However, individuals with secondary education, primary education, and illiteracy are respectively 1.42, 1.87, and 8.60 times more likely to experience inaccessible times to the dialysis unit when compared to those with a diploma education level or above (Table 5).

**Table 5:** Factors associated with missing dialysis sessions

Variable	Category	Missed dialysis session in the past year		*p* value	Odds ratio (95% CI)	Binary logistic analysis *p* value
		*No Freq (%)*	*Yes Freq (%)*			
*Education Level*	Diploma and above (Ref)	10 (9.9%)	1 (1.0%)	0.02		
	Secondary	11 (10.9%)	3 (3%)		1.417 (0.107–18.745)	0.791
	Primary	37 (36.6%)	11 (10.9%)		1.866 (0.189–18.434)	0.594
	Illiterate	14(13.9%)	14 (13.9%)		8.579 (0.837–87.932)	0.07
*Residency Status*	Displaced	29 (28.7%)	19 (18.8%)	0.02		
	Host community– not displaces	43 (42.6%)	10 (9.9%)			
*Frequency of residency relocation due to insecurity*	No relocation	34 (33.7%)	6 (5.9%)	0.04		
	Once	10 (9.9%)	3 (3.0%)			
	Twice	11 (10.9%)	8 (7.9%)			
	Three times	8 (7.9%)	4 (4.0%)			
	Four times	6 (5.9%)	2 (2.0%)			
	More than four times	3 (3.0%)	6 (5.9%)			
*Moving Due to Insecurity*	Yes	38 (37.6%)	23 (22.8%)	0.01	4.5 (1.399–14.477)	0.012*
	No	34 (33.7%)	6 (5.9%)			
*Utilization of hemodialysis services from multiple units in the past year*	Yes	17 (16.8%)	16 (16.9%)	0.002	3.824 (1.401–10.44)	0.009*
	No (Ref)	55 (54.4%)	13 (12.9%)			
*Regular dialysis locations over the past year*	One location	55 (54.4%)	13 (12.9%)	0.01		
	Two locations	10 (9.9%)	11 (10.9%)			
	Three locations	7 (6.9%)	5 (5.0%)			

*Statistically significant

## Discussion

This survey offers important insights into the experiences and challenges faced by patients with ESKD in conflict zones, particularly regarding dialysis. health literacy, access and continuity. Our findings underscore a significant disruption in dialysis treatment, primarily due to the instability and frequent relocations caused by ongoing conflict. Notably, 28.7% of the patients encountered interruptions in their dialysis sessions, which could potentially worsen their health outcomes. Our study is one of the few that have explored the perspectives of ESKD patients and the barriers they face in accessing care and impact of their socioeconomic status and dialysis literacy on their access in conflict situations.

The substantial number of IDPs within the study cohort highlights the mobility forced upon these patients by conflict. This forced migration has a direct association with the frequency of missed dialysis sessions. Our data suggests that patients who remain stationary are less likely to miss treatments compared to those who have moved multiple times [3, 10, 11]. A quarter of patients had issues requiring moving households. Around 34% of these moves were due to perceived insecurity. There was also an element in the irregularity of attendance at regular dialysis sessions reported by 29 (28.7%) patients. In some cases, it was the facility itself that was closed for insecurity rather than patients feeling travel was unsafe. Information on facility closure and accessible alternative facilities was communicated by WhatsApp groups from dialysis facilities and the health directorates. Those patients who had not been frequently displaced missed fewer sessions (*p* = 0.022). This is consistent with findings in non-conflict areas [12]. In NW Syria various logistical factors, such as transportation costs, mode of travel to the current dialysis, estimated travel duration to the nearest dialysis unit, and duration from home to the current dialysis site, did not show any statistically significant associations with missing dialysis session in the past year.

Information on dialysis schedule compliance in other conflict situations have not been reported. However, in non-conflict situations in low- and middle-income countries, missing scheduled sessions has ranged from 35.8 to 51% in a year [13–17]. In these reports there were no clear statistical association between visit compliance and factors relating to age, sex, and the economic factors including occupation status and income. Other studies have shown that adherence may be influenced by sex and age, with non-adherence being more common among younger patients [16–18]. There have been suggestions that low formal education levels are associated with low health literacy which may in turn lead to irregular health compliance, a pattern we observed in the NW Syria sample [19]. Overall literacy rates in Syria was reported as 94% [20]. Among the displaced and conflict affected population in NW Syria there are reasons such as an older population and extensive regional disturbances of civil society, which could contribute to a lower literacy rate.

As expected of a population with many IDPs in a conflict area, there was movement by patients among the hemodialysis units. While 67.3% of persons used the same hemodialysis unit throughout the previous year, 20.8% had used two sites in the year and 11.9% reported using three sites. While it is important that there are reliable treatment point options for care at present there is no system to easily share patient records among facilities when security deteriorated or other events blocked access to usual dialysis sites [21]. 

Despite logistical efforts to facilitate access, including the strategic placement of dialysis units to reduce travel time, patients still face significant barriers. These include the physical risks of traveling in a conflict zone and the interrupted operation of dialysis centers due to security concerns. The data reveals that while many patients can reach a dialysis center within an hour, the unpredictability of service availability remains a critical issue. Most patients (81.1%) reported a travel time to their usual hemodialysis unit of less than 60 min. Even when alternative sites had to be used, the distances were manageable by patients. This was probably a factor in less than half (42.6%) reporting out-of-pocket costs for transport to a dialysis unit. The association between proximity and use of dialysis services has been noted elsewhere [22].

Only about half of hemodialysis patients reported being seen by a doctor in the past six months, and of these, only 48.5% said they received specific feedback from their doctors concerning their dialysis treatment plan. Patients did report having vital signs checked at each dialysis. Furthermore, our study indicates a concerning gap in health literacy regarding ESKD and dialysis among patients. Only 32.7% of participants showed a comprehensive understanding of their condition and treatment, which is alarming, considering the complexity of their needs. This lack of awareness could impede effective disease management and exacerbate health risks. In their assessments of dialysis programs in NW Syria, the hemodialysis taskforce found patient education activities weak [3]. Given the shortage of physicians and nephrologists in this conflict area, coupled with the high prevalence of comorbidities, high dialysis literacy guiding self-management is particularly important for this population.

The findings highlight gaps in dialysis care in Northwest Syria that NGOs and healthcare providers can address. The lack of regular physician oversight for hemodialysis patients suggests an urgent need for structured follow-up protocols. Additionally, low patient health literacy regarding their disease and treatment underscores the necessity of dialysis facility-based education programs tailored to conflict settings. Service disruptions due to facility closures and insecurity further emphasize the importance of improving coordination between dialysis centers, potentially through shared patient records or contingency plans for displaced patients. Expanding free transportation or mobile dialysis units could also enhance access for those facing travel barriers. These targeted interventions could significantly improve dialysis care in resource-limited conflict zones.

The work by the hemodialysis taskforce in NW Syria with support from the WHO, has conducted training based on initial performance assessments, set performance standards and established a regular supervision and assessment routine [10, 23]. While these have clearly improved the quality of services and infection control procedures, there are still deficits related to movement of supplies and maintaining the technical skills needed to care for ESKD patients in a conflict situation. Kidney transplantation is an option to be considered in some conflict situations [24]. Although not suitable in NW Syria at the time of this study, with the recent fall of the Assad regime this could become feasible However, even if transplantation does become more widely available. more work is needed to mitigate the challenges faced by ESKD patients in conflict zones. This survey showed that a multifaceted approach is required. First, strengthening the infrastructure of hemodialysis services through international collaboration and local partnerships can ensure that facilities remain operational even during periods of intensified conflict. This includes securing supply chains for dialysis materials and safeguarding access routes to dialysis centers. Second, enhancing patient education programs to improve health literacy is critical. These programs should be culturally tailored and accessible, perhaps utilizing mobile technology to reach patients in remote or insecure areas. Additionally, integrating ESKD care into the broader emergency response plans of health agencies and non-governmental organizations could ensure that dialysis services are prioritized alongside acute care provisions. Finally, fostering community support networks can provide emotional and logistical support to help patients navigate the challenges of accessing care. By adopting these strategies, we can build a more resilient healthcare system that not only withstands the challenges of conflict but also promotes the long-term well-being of vulnerable populations.

Dialysis care in conflict zones faces persistent challenges due to supply chain disruptions, infrastructure damage, lack of medical oversight, and severe healthcare workforce shortages. Evidence from recent conflicts further highlights these gaps. A study on hemodialysis patients during the Sudanese civil conflict found that limited access to dialysis supplies, frequent service interruptions, and inadequate medical oversight were major barriers, mirroring the situation in Northwest Syria [25–27]. In Ethiopia’s Tigray conflict, dialysis services were nearly eliminated, with over 250 dialysis patients dying due to prolonged supply shortages.3 In Ukraine, conflict-driven disruptions forced facilities to reduce dialysis session frequency and duration, increasing patient morbidity [27, 28]. Meanwhile, Lebanon’s economic collapse has severely impacted dialysis access, leading to facility closures and financial barriers for patients [29]. The Syrian crisis further illustrates these challenges, with northwest Syria’s dialysis centers relying on fragmented NGO funding, resulting in inconsistent care quality, inadequate infection control, and limited access to lab testing and nephrology expertise [10, 27]. 

This study has limitations. We relied on phone interviews with ESKD patients, which excluded several people who were unable to participate for connectivity reasons. In some cases, both caregivers and patients provided information, which may have introduced some bias. Two dialysis units declined to provide access to patients using their facilities, and the exclusion of these limits the representativeness of the study. These non-participating clinics and their clients did not differ in specific characteristics from those where patients did take part, suggesting that their absence did not introduce biases. Yet the randomized systematic sampling approach used did provide a 11.9% sample of dialysis patients in NW Syria which we believe is representative.

## Conclusion

This survey underscores the resilience of both ESKD patients and dialysis units in conflict-affected areas but also exposes significant gaps in healthcare and patient education. The ongoing conflict has disrupted access to dialysis facilities, either by making them unreachable or causing their closure. This situation is exacerbated by a shortage of specialist nephrologists, which limits effective patient care and reduces health literacy about ESKD management.

Addressing these challenges requires collaboration among healthcare providers, humanitarian agencies, and policymakers to ensure uninterrupted care. Steps toward improving this situation include enhancing healthcare infrastructure, optimizing dialysis service operations, and increasing patient education on ESKD. Additionally, employing internists and general physicians for interim management could improve care, while expanding the role of dialysis technicians to boost patient dialysis literacy might offer further benefits. These efforts are crucial for building a resilient healthcare system that supports patient well-being in conflict settings.

## Supplementary Information

Supplementary Material 1.

## Data Availability

The data for this study are available by application to Ismail Alkhatib at drsmile86@gmail.com.

## References

[1] Aebischer PS, Martinez E, du Mortier S, Rossi R, Pahud M, Urbaniak V, Chappuis F, Hagon O, Jacquérioz BF, Beran D. Non-communicable diseases in humanitarian settings: ten essential questions. Confl Health. 2017;11:17. 10.1186/s13031-017-0119-8. PMID: 28932259; PMCID: PMC5602789. 28932259 10.1186/s13031-017-0119-8 PMC5602789

[2] Oni T, Unwin N. Why the communicable/non-communicable disease dichotomy is problematic for public health control strategies: implications of Multimorbidity for health systems in an era of health transition. Int Health. 2015;7(6):390–9. 10.1093/inthealth/ihv040. Epub 2015 Jun 23. PMID: 26103981; PMCID: PMC4638105. 26103981 10.1093/inthealth/ihv040 PMC4638105

[3] Alasfar S, Alashavi H, Nasan KH, Haj Mousa AA, Alkhatib I, Kazancioglu R, Sekkarie M, Kaysi S, Daher M, Murad L, Burnham GM. Improving and maintaining quality of hemodialysis in areas affected by war: a call to action! Kidney Int. 2023;103(5):817–20. 10.1016/j.kint.2023.02.004. PMID: 37085252. 37085252 10.1016/j.kint.2023.02.004

[4] Syrian Arab Republic: 2023 Humanitarian Needs Overview (December 2022) - Syrian Arab Republic| ReliefWeb. https://reliefweb.int/report/syrian-arab-republic/syrian-arab-republic-2023-humanitarian-needs-overview. Accessed 28 Jul 2024.

[5] Alhaffar MHDBA, Janos S. Public health consequences after ten years of the Syrian crisis: a literature review. Global Health. 2021;17(1):111. 10.1186/s12992-021-00762-9. PMID: 34538248; PMCID: PMC8449996. 34538248 10.1186/s12992-021-00762-9 PMC8449996

[6] Sekkarie MA, Zanabli AR, Rifai AO, Murad LB, Al-Makki AA. The Syrian conflict: assessment of the ESRD system and response to hemodialysis needs during a humanitarian and medical crisis. Kidney Int. 2015;87(2):262–5. 10.1038/ki.2014.336. PMID: 25635715. 25635715 10.1038/ki.2014.336

[7] Moukeh G, Yacoub R, Fahdi F, Rastam S, Albitar S. Epidemiology of hemodialysis patients in Aleppo city. Saudi J Kidney Dis Transpl. 2009;20(1):140–6 PMID: 19112237. 19112237

[8] Sekkarie M, Murad L, Al-Makki A, Al-Saghir F, Rifai O, Isreb M. End-stage kidney disease in areas of armed conflicts: challenges and solutions. Semin Nephrol. 2020;40(4):354–62. 10.1016/j.semnephrol.2020.06.003. PMID: 32800286. 32800286 10.1016/j.semnephrol.2020.06.003

[9] North-West Syria. Situation Report (15 March 2023). ReliefWeb. https://reliefweb.int/report/syrian-arab-republic/north-west-syria-situation-report-15-march-2023-enar. Accessed 28 Jul 2024.

[10] Alasfar S, Alashavi H, Nasan KH, Haj Mousa AA, Polinori C, Luyckx V, Sekkarie M, Kaysi S, Murad L, Burnham GM. Providing Hemodialysis in unstable areas: an assessment and framework for effective care. Kidney Int Rep. 2023;9(3):580–8. PMID: 38481490; PMCID: PMC10927480. 38481490 10.1016/j.ekir.2023.12.006 PMC10927480

[11] Koubar SH, Hajj Nasan K, Sekkarie MAK. Nephrology workforce and education in conflict zones. Kidney Int Rep. 2021;7(2):129–32. 10.1016/j.ekir.2021.11.024. PMID: 35155849; PMCID: PMC8820984. 35155849 10.1016/j.ekir.2021.11.024 PMC8820984

[12] Genereux D, Fan L, Brownlee K. The psychosocial and somatic effects of relocation from remote Canadian first Nation communities to urban centres on Indigenous peoples with chronic kidney disease (CKD). Int J Environ Res Public Health. 2021;18(7):3838. 10.3390/ijerph18073838. PMID: 33917560; PMCID: PMC8038784. 33917560 10.3390/ijerph18073838 PMC8038784

[13] Mohamedi S, Mosha IH. Hemodialysis therapy adherence and contributing factors among End-Stage renal disease patients at muhimbili National hospital, Dar Es Salaam, Tanzania. Kidney Dialysis. 2022;2(1):123–30. 10.3390/kidneydial2010014.

[14] AlKhattabi G. Prevalence of treatment adherence among attendance at Hemodialysis in Makah. Int J Med Sci Public Health. 2014;3(5):592. 10.5455/IJMSPH.2014.170320141.

[15] Naalweh KS, Barakat MA, Sweileh MW, Al-Jabi SW, Sweileh WM, Zyoud SH. Treatment adherence and perception in patients on maintenance hemodialysis: a cross - sectional study from Palestine. BMC Nephrol. 2017;18(1):178. 10.1186/s12882-017-0598-2. PMID: 28558719; PMCID: PMC5450383. 28558719 10.1186/s12882-017-0598-2 PMC5450383

[16] Mukakarangwa MC, Chironda G, Bhengu B, Katende G. Adherence to Hemodialysis and associated factors among end stage renal disease patients at selected nephrology units in Rwanda: A descriptive Cross-Sectional study. Nurs Res Pract. 2018;2018:4372716. 10.1155/2018/4372716. PMID: 29973988; PMCID: PMC6008892. 29973988 10.1155/2018/4372716 PMC6008892

[17] Alzahrani A, Al-Khattabi AMGH. Factors influencing adherence to hemodialysis sessions among patients with end-stage renal disease in Makkah City. Saudi J Kidney Dis Transpl. 2021;32(3):763–73. 10.4103/1319-2442.336772. PMID: 35102919. 35102919 10.4103/1319-2442.336772

[18] Halle MP, Nelson M, Kaze FF, Jean Pierre NM, Denis T, Fouda H, Ashuntantang EG. Non-adherence to Hemodialysis regimens among patients on maintenance Hemodialysis in sub-Saharan Africa: an example from Cameroon. Ren Fail. 2020;42(1):1022–8. PMID: 33028122; PMCID: PMC7580605. 33028122 10.1080/0886022X.2020.1826965 PMC7580605

[19] Paasche-Orlow MK, Parker RM, Gazmararian JA, Nielsen-Bohlman LT, Rudd RR. The prevalence of limited health literacy. J Gen Intern Med. 2005;20(2):175–84. 10.1111/j.1525-1497.2005.40245.x. PMID: 15836552; PMCID: PMC1490053. 15836552 10.1111/j.1525-1497.2005.40245.x PMC1490053

[20] The Global Economy.com. Syria: Literacy rate. https://www.theglobaleconomy.com/Syria/literacy_rate/. Accessed 20 Feb 2025.

[21] Tong A, Manns B, Hemmelgarn B, Wheeler DC, Evangelidis N, Tugwell P, Crowe S, Van Biesen W, Winkelmayer WC, O’Donoghue D, Tam-Tham H, Shen JI, Pinter J, Larkins N, Youssouf S, Mandayam S, Ju A, Craig JC. SONG-HD investigators. Establishing core outcome domains in hemodialysis: report of the standardized outcomes in Nephrology-Hemodialysis (SONG-HD) consensus workshop. Am J Kidney Dis. 2017;69(1):97–107. 10.1053/j.ajkd.2016.05.022. Epub 2016 Aug 3. PMID: 27497527; PMCID: PMC5369351. 27497527 10.1053/j.ajkd.2016.05.022 PMC5369351

[22] Gulliford M, Figueroa-Munoz J, Morgan M, Hughes D, Gibson B, Beech R, Hudson M. What does ‘access to health care’ mean? J Health Serv Res Policy. 2002;7(3):186–8. 10.1258/135581902760082517. PMID: 12171751. 12171751 10.1258/135581902760082517

[23] Burnham G, Alashavi H, Alasfar S. Quality improvement methods to strengthen Hemodialysis in Northwest Syria. J Glob Health Sci. 2022. 10.35500/jghs.2022.4.e16.

[24] Alasfar S, Isreb M, Kaysi S, Hatahet K. Renal transplantation in areas of armed conflict. Semin Nephrol. 2020;40(4):386–92. 10.1016/j.semnephrol.2020.06.006. PMID: 32800289. 32800289 10.1016/j.semnephrol.2020.06.006

[25] Idrees MHD, Bashir MMI, Mohamed BAA, Ahmed AEA, Abdalla HMM, Shaaban KMA. April 15th war and Hemodialysis patients in Sudan: a cross-sectional study. BMC Public Health. 2025;25(1):230. 10.1186/s12889-025-21369-4. PMID: 39833802; PMCID: PMC11744938. 39833802 10.1186/s12889-025-21369-4 PMC11744938

[26] Sekkarie M, Murad L, Alasfar S. Assessment of the response to kidney patients’ needs in disaster-stricken Syria. Curr Opin Nephrol Hypertens. 2024;33(6):621–6. 10.1097/MNH.0000000000001009. Epub 2024 Jun 20. PMID: 38900090. 38900090 10.1097/MNH.0000000000001009

[27] Alasfar S, Koubar SH, Gautam SC, Jaar BG. Kidney care in times of crises: A review. Am J Kidney Dis. 2024;84(5):621–31. 10.1053/j.ajkd.2024.03.030. Epub 2024 Jun 6. PMID: 38851445. 38851445 10.1053/j.ajkd.2024.03.030

[28] Stepanova N, Kolesnyk M, Mithani Z, Alkofair B, Shakour RL, Petrova A, Novakivskyy V, Hymes JL, Brzosko S, Giullian J, Espinel Z, Shultz JM. Lifesaving care for patients with kidney failure during the war in Ukraine 2022. Clin J Am Soc Nephrol. 2022;17(7):1079–81. Epub 2022 May 10. PMID: 35537755; PMCID: PMC9269632. 35537755 10.2215/CJN.04720422 PMC9269632

[29] Alasfar S, Berhe E, Karam S, Luyckx V. Impact of persistent conflict and destabilizing events on dialysis care. Nat Rev Nephrol. 2023;19(11):688–9. 10.1038/s41581-023-00759-0. PMID: 37587370. 37587370 10.1038/s41581-023-00759-0

