# Supplementary material for: End-stage kidney diseases in areas of conflict: patients’ perspective and patient access to hemodialysis services in Northwest Syria
Source: BMC Health Serv Res. 2025 May 2;25:638. doi: 10.1186/s12913-025-12673-1 (PMC12046966; doi:10.1186/s12913-025-12673-1)
Supplement: Supplementary file 1 — Supplementary Material 1. [file 12913_2025_12673_MOESM1_ESM.pdf]

End-Stage Kidney Diseases in Areas of Conflict: Patients' Perspective and Access to Hemodialysis Services in Northwest Syria

Study Questionnaire:

Date_____, interviewer code ______, interview number______

☐ patient interviewed ☐ someone answered on behalf of the patient.

Number	Question	Response	skip
**A. Demographic information**			
A1	What is your age?	years_________	
A2	What is your sex?	M____ F___	
A3	Marital status?	Single – married - divorced	
A4	Education level?	Illiterate<br>Primary<br>Secondary<br>Diploma and above	
A5	How many years of school have you completed?	years _________	
A6	How many adults and children under 18 live in your house	Adults_____<br>Children under 18 _____	
A7	From where do you originally come?		
A8	Where do you live now?		
A9	How many times have you moved because of conflict	times moved_______	
**B. Employment**			
B1	Are you working now?	Yes or No	
B2	If Yes, What is your current occupation?	Farmer<br>Employee<br>Health worker<br>Daily labor worker<br>others	
B3	If work, how would you describe your work?	Full time ....1<br>Half time..................................2<br>Less than half time...................3<br>Full time housewife ....4<br>Retired or disabled...................5	
B4	How many people in your household are earning money regularly?		
**C. Medical History**			
C1	How many years ago did you first know you had kidney disease?		
C2	How many years ago did you start hemodialysis?		
C3	What other medical conditions has a doctor told you are present?	High blood pressure................1<br>Diabetes (no insulin) ....2<br>Diabetes (insulin) ....3<br>Heart Disease ....4<br>Stroke ....5<br>Other (list) 6	

**D. Dialysis**			
D1	Most weeks how many dialysis sessions do you have in a week?	Dialysis sessions_____	
D2	In the past year what locations have your regularly (more than 1 wk) received dialysis?	Number of places_______	If 0 ➔
D3	If >1 why did you change to the current location	Household moved from conflict .... 1<br>Household moved for other reasons .... 2<br>Dialysis unit closed .... 3<br>Another unit more convenient .... 4<br>Others (list) 5	
D4	How long does take to reach current dialysis site from your home?	Hours ______________	
D5	How do you travel to this site?	My car/ motorcycle................. 1<br>Friend or other drives me....... 2<br>Taxi/ public transportation...... 3<br>Other (list) 4	
D6	Do you pay someone to take you for dialysis	Yes..1<br>No ....2<br>Varies—sometimes................3	If NO ➔
D7	If so how much did you pay	Lira________<br>Dollars ______	
D8	Have there been times in past year when you could not reach the dialysis unit?	Yes..1<br>No 2	If NO ➔
D9	If YES, how often	Once a week ………………….1<br>Once a month…………………2<br>Once every few months………3<br>Less often than above ……….4	
D10	If YES, what were the reasons?	Insecurity ....1<br>No transport...........................2<br>Too sick to travel....................3<br>Dialysis unit closed ....4<br>Other (List).............................5	

D11	If something happened now that blocked coming to this unit for dialysis, what would you do?	*describe*	
D12	If something happened now that blocked coming to this unit for dialysis, how long will it take you to reach the next nearest dialysis center?	….hours	
**E. Present treatment facility**			
E1	For how long have you been getting dialysis at the present health facility?	Months _______ or<br>Years_________	
E2	During your dialysis sessions, do you get the following?	Weighed (your weight is measured) YES/NO<br>Blood pressure taken during dialysis YES/NO<br>Examined for signs of swelling or oedema YES/NO<br>Listened to my chest with stethoscope (chest auscultation) YES/NO<br>told my blood was taken for lab test YES/NO<br>Given health advice YES/NO<br>Other (LIST)	
**F. Health /dialysis literacy**			
F1	Which of the following is NOT a purpose of dialysis?	A. To remove excess bodily fluid<br>B. To filter out metabolic waste from the blood<br>C. To improve the function of the digestive system<br>D. To balance the body's electrolytes	
F2	Which of the following statements regarding artificial blood vessel (Port-A) or arteriovenous fistula care is TRUE?	A.<br>The patient can touch it or check with a stethoscope—it is functioning normally if it is silent.<br>B.<br>The patient's blood pressure can be measured on the arm with the fistula.<br>C.<br>A warm compress should be applied to the site of the fistula to induce blood circulation on the day of dialysis.<br>D.	

		Redness at the site is normal and is no cause for concern.	
F3	When last time checked by doctor?	Month/year _______	
F4	What did the doctor tell you about your dialysis?	Nothing .... 1<br>Don't remember .... 2<br>Specifics (list)........................ 3	
F5	Last time had blood tests?	Months ago____________	
F6	Can you remember what test it was?	No .... 1<br>Yes (describe)......................... 2	
F7	When you have a lab test done, does the staff tell you the results during your next visits?	Yes..1<br>No ....2<br>Sometimes ....3<br>Don't remember 4	

End of the Questionnaire

Thank the patient very much for taking the time to talk with you.
